# Neutrophils and polymorphonuclear myeloid-derived suppressor cells: an emerging battleground in cancer therapy

**DOI:** 10.1038/s41389-022-00398-3

**Published:** 2022-05-03

**Authors:** Hans Raskov, Adile Orhan, Shruti Gaggar, Ismail Gögenur

**Affiliations:** 1 Center for Surgical Science, Zealand University Hospital, Køge, Denmark; 2 Department of Biomedical Sciences, University of Copenhagen, Copenhagen, Denmark; 3 Department of Clinical Medicine, University of Copenhagen, Copenhagen, Denmark

**Keywords:** Cancer microenvironment, Tumour immunology

## Abstract

Neutrophils are central mediators of innate and adaptive immunity and first responders to tissue damage. Although vital to our health, their activation, function, and resolution are critical to preventing chronic inflammation that may contribute to carcinogenesis. Cancers are associated with the expansion of the neutrophil compartment with an escalation in the number of polymorphonuclear myeloid-derived suppressor cells (PMN-MDSC) in the peripheral circulation and tumor microenvironment. Although phenotypically similar to classically activated neutrophils, PMN-MDSC is pathologically activated and immunosuppressive in nature. They dynamically interact with other cell populations and tissue components and convey resistance to anticancer therapies while accelerating disease progression and metastatic spread. Cancer-associated neutrophilia and tumor infiltration of neutrophils are significant markers of poor outcomes in many cancers. Recently, there has been significant progress in the identification of molecular markers of PMN-MDSC providing insights into the central role of PMN-MDSC in the local tumor microenvironment as well as the systemic immune response in cancer. Further advances in sequencing and proteomics techniques will improve our understanding of their diverse functionalities and the complex molecular mechanisms at play. Targeting PMN-MDSC is currently one of the major focus areas in cancer research and several signaling pathways representing possible treatment targets have been identified. Positive results from preclinical studies clearly justify the current investigation in drug development and thus novel therapeutic strategies are being evaluated in clinical trials. In this review, we discuss the involvement of PMN-MDSC in cancer initiation and progression and their potential as therapeutic targets and clinical biomarkers in different cancers.

## Introduction

Malignant cells are able to escape immune surveillance mainly by escalating the production of pathologically activated myeloid-derived suppressor cells (MDSC). It is quite evident that cytotoxic T cells, memory T cells, T_H_1 cells, and B cells are associated with prolonged survival, whereas elevated densities of T_reg_ cells, MDSC, and neutrophils are with poor prognosis (reviewed in [1]). Notably, in multiple solid and non-solid tumors, the number of circulating MDSC positively correlates to cancer stage and shorter survival [2–5].

**Fig. 1:**
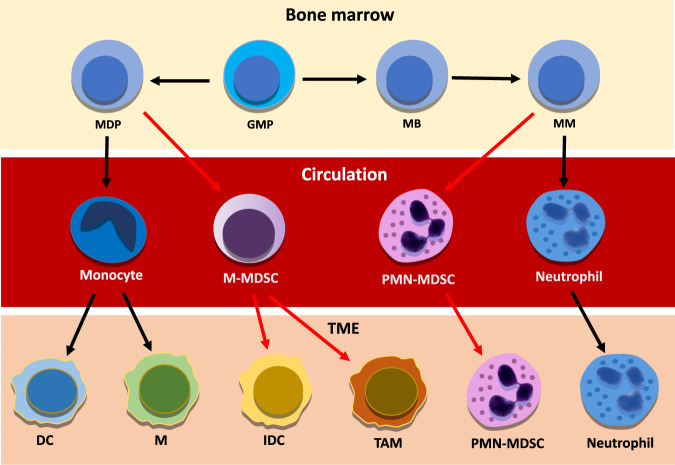
Illustrates the differentiation of neutrophils and myeloid-derived suppressor cells from bone marrow to bloodstream and tumor microenvironment. Red arrows indicate pathological activation. Within the tumor microenvironment, classically activated neutrophils hold antitumor functions, whereas immunosuppressive polymorphonuclear myeloid-derived suppressor cells drive tumor-promoting inflammation associated with poor oncological outcomes. The majority of monocytic myeloid-derived suppressor cells differentiate into TAM and inflammatory dendritic cells contributing to immune suppression and chronic inflammation, DC dendritic cell, GMP granulocyte-macrophage progenitor, IDC inflammatory dendritic cell, M macrophage, MB myeloblast, MDP monocyte/macrophage/dendritic cell progenitor, MM meta-myelocyte, M-MDSC monocytic myeloid-derived suppressor cell, PMN-MDSC, polymorphnuclear myeloid-derived suppressor cell, TAM tumor-associated macrophage. TME tumor microenvironment.

Under a constant influence of tumor-secreted growth factors and inflammatory mediators, myelopoiesis derails and polymorphonuclear (PMN)-MDSC and monocytic population of MDSC (M-MDSC) expand heavily from their precursors in the bone marrow (Fig. 1). Following chemokine concentration gradients, MDSC is drawn into circulation and to the tumor microenvironment (TME) where they support the tumor development primarily by suppressing the cytotoxic anticancer immune responses.

When in the TME, most M-MDSC differentiate into other cell types (see below), whereas polymorphonuclear myeloid-derived suppressor cells (PMN-MDSC) amplify their pro-tumorigenic immunosuppressive capacities and resistance to apoptosis due to a phenotypic switch mediated by the TME [6, 7].

In the early stages of malignant transformation, classically activated antitumor neutrophils are prevalent. However, during disease progression, PMN-MDSC levels increase to become a prominent immunosuppressive cell type in the circulation from where they are recruited to the TME to aid in the establishment of an immunosuppressive milieu that facilitates tumor escape [8, 9].

Following the arrival in the TME, PMN-MDSC amplify their pro-tumorigenic immunosuppressive capacities and resistance to apoptosis due to a phenotypic switch mediated by the TME [6, 7], whereas M-MDSC, differentiate into tumor-associated macrophages (TAM), dendritic cells, and fibrocytes [10–13].

Although once considered a short-lived and static cell population, it is now clear that the extended lifespan and pronounced plasticity of neutrophils are highly dictated by the environment [14]. Within the TME, classically activated neutrophils hold antitumor functions, whereas immunosuppressive PMN-MDSC drives tumor-promoting inflammation that has been associated with poor oncological outcomes [15].

Due to their strong phenotypical and morphological similarities, most previous studies have investigated circulating neutrophils and tumor-associated neutrophils (TAN) without distinguishing between the classically activated neutrophils and the pathologically activated PMN-MDSC, suggesting that the individual contribution of PMN-MDSC in cancer progression has been not fully explored.

The varied nomenclature used in published research for immunosuppressive neutrophils include TAN, N2 neutrophils, or N2 cells (with reference to the former macrophage nomenclature), immature neutrophils, alternatively activated neutrophils, low-density neutrophils, and granulocytic- or G-MDSC.

To simplify the nomenclature in this review, we use the term neutrophils for classically activated antitumor neutrophils; PMN-MDSC for pathologically activated, pro-tumorigenic immunosuppressive neutrophils; and PMN-MDSC^TME^ for the TME-infiltrating subset of PMN-MDSC. We discuss the role of PMN-MDSC and PMN-MDSC^TME^ in cancer and as predictors and therapeutic targets. Referring to previous TME-studies without the distinction between neutrophils and PMN-MDSC^TME^, the term TAN has been used. Likewise, previous studies on the prognostic capacity of the neutrophil-to-lymphocyte ratio (NLR) do not discriminate between immunocompetent neutrophils and MDSC and thus, the term neutrophil is retained in the NLR section. Nonetheless, PMN-MDSC as a cell type is a poorly defined concept and the definitions used here are not canonical. We acknowledge that other definitions exist, including previous marker-based definitions. To this end, the term “pathologically activated” does not bear any different functional meaning than immunosuppressive in contrast to classically activated, immunocompetent neutrophils.

## Identification of neutrophils and PMN-MDSC

Even though MDSC play beneficial roles in pregnancy to maintain the maternal-fetal tolerance and in sepsis, trauma, and surgery to balance the powerful inflammatory responses; MDSC are most harmful in malignancy owing to their immunosuppressive activities. The expansion of the MDSC compartment during the postoperative surgical stress response may have detrimental effects on the clinical outcome of cancer [16].

Neutrophilia is highly prevalent among patients with cancer. Using gene expression profiles from more than 18,000 patients, the CIBERSORT study showed that TAN was the single-cell population most highly associated with mortality for multiple cancers [17].

In a recent study on healthy donors and cancer patients, classically activated neutrophils were identified as CD11b^+^CD15^+^CD16^+^CD66b^high^Arg1^+/−^STAT3^-^S100A9^+^LOX^−^ and PMN-MDSC in the peripheral blood from patients with NSCLC were identified as CD11b^high^CD15^high^CD66b^high^CD33^high^Arg1^high^S100A9^high^Lox1^high^ [18]. The PMN-MDSC contains high levels of prostaglandin E2 (PGE2), ROS, inducible nitric oxide synthase, and arginase [19]. A high expression of arginase diminishes T cell CD3ζ expression and impairs the aggregation of the T cell receptor. The lectin-type oxidized LDL receptor 1 (LOX1), a specific marker of PMN-MDSC, is associated with abnormal lipid metabolism [20]. Recently, the fatty acid transport protein 2 (FATP2) also was identified as a specific PMN-MDSC marker [21].

Although PMN-MDSC should be subjected to a co-culture T cell assay to determine their immunosuppressive activity, the progress in the identification of molecular markers represents a significant advance in the classification of these cells [7, 18]. Nevertheless, the surface markers expressed by MDSC mostly overlap with those conventionally used to identify monocytes and neutrophils. Further, it is still debated whether MDSC should be regarded as a separate entity or rather as monocytes or neutrophils affected by immunosuppressive tumor-derived factors [22].

Studies on the myeloid cell landscape of tumors have confirmed a significant heterogeneity of myeloid cells in cancer using microscopy and flow cytometry. Single-cell RNA sequencing and mass cytometry demonstrated a diversity among innate immune cells corresponding to immature PMN-MDSC from various stages of myelopoiesis and to differentially activated mature neutrophils. It appears that immunosuppressive PMN-MDSC coexists not only with the classical neutrophils but also with less activated and less suppressive PMN-MDSC (reviewed in [23]).

In addition, single-cell RNA sequencing in mice models has confirmed the co-existence of classical neutrophils and PMN-MDSC in tumors, in which PMN-MDSC appeared very early in tumor development and remained the most abundant PMN neutrophil population [18].

## Neutrophils

Along with other cell types of the innate immune system—such as natural killer cells, macrophages, and dendritic cells—neutrophils constitute the first-line of cellular defense against invading microbes and malformed host cells. During homeostasis, neutrophils are produced in numbers of 10^11^ cells/day in humans, representing 50–70% of the circulating leukocytes and the largest immune-cell population in circulation. In addition, neutrophils are present in healthy tissues with striking tissue-driven heterogeneity in their functions across different organs [24].

Primarily regulated by the microbiota, neutrophils survive for a few hours to a few days and require a constant replenishment from their granulocyte-monocyte precursors in the bone marrow [25]. A study using deuterium oxide-labeled human neutrophils estimated neutrophil half-life to be up to 5.4 days in vivo [26], which contrasts with the previously estimated half-life of less than one day based on ex vivo labeling [27].

Specifically, in a mouse model of head and neck cancer, neutrophils infiltrated a tumor transplant within a few hours and persisted in the TME for up to 3 days [28]. At sites of cancer and inflammation, the exposure to cytokines such as the interleukin 1β (IL1β) and granulocyte-colony-stimulating factor (G-CSF) and the recognition of neutrophil pattern recognition receptors (PRR) by damage-associated molecular patterns (DAMP) and pathogen-associated molecular patterns (PAMP) increase the neutrophil lifespan dramatically through inhibition of apoptosis [29].

In response to external physical and chemical properties in the local environment, neutrophils make frequent shifts in functionality and express an arsenal of cytokines and proteolytic enzymes [30] that are stored in their granules [31]. However, neutrophil effector functions maintaining tissue health may also participate in the initiation and progression of malignant transformation such as modeling of extracellular matrix (ECM), angiogenesis, and release of neutrophil extracellular traps (NET) that may aid in the migration and dissemination of tumor cells [32–35].

### Neutrophil life cycle

A fine balance between production, retention, mobilization, and clearance tightly regulates the number of circulating neutrophils. While the α-C-X-C chemokine receptor type 4 (CXCR4) signaling retains the neutrophils in the bone marrow, the G-CSF signaling promotes the survival, proliferation, and differentiation of granulocytic progenitor cells. Further, the expression of G-CSF and interaction of IL8 with CXCR1/2 subsequently drive the mature neutrophils into circulation [36]. In inflammation, powerful inflammatory mediators such as CXC chemokines, tumor necrosis factor-alpha (TNFα), leukotriene B4, IL8, and complement component 5a rapidly mobilize neutrophil swarms (resembling the characteristics of swarming insects), during which multiple neutrophils coordinate and enhance their effector functions synergistically and aggregate on the target [37].

Fascinatingly, the release of neutrophils and their clearance are synchronized by a physiological circadian rhythm that releases mature neutrophils in the early morning and in the night [38]. Migrating towards concentration gradients of the C-X-C motif chemokine ligand 12 (CXCL12 - a CXCR4 ligand), aged or exhausted neutrophils return from their field of action to be phagocytized by macrophages in the bone marrow, spleen, liver, and lungs. In response to this clearance, macrophages release G-CSF that in addition to stimulating the production and release of new neutrophils into the bloodstream, also enhances the neutrophil effector functions [39].

The ligation of the neutrophil CXCR1/2 receptors by chemotactic and angiogenic factors mediates the activation and migration of neutrophils to the target areas. When penetrating the tissues, neutrophils may switch chemoattractant preferences and functional phenotypes [40]. Although the target tissues are regarded as the main chemokine producers, neutrophils also produce these chemokines to amplify the recruiting signals.

Human neutrophils express β_1_, β_2_, and β_3_ integrins (cluster of differentiation [CD]-29, CD18, and CD61) that recognize ECM components (fibronectin, fibrinogen, and collagen) and identify locations for intravascular adhesion and transmigration from circulation to the target area [41]. Once engaged by their ligands, the conformation of these integrins changes to secure the anchoring to the endothelial membrane and the activation of reactive oxygen species (ROS) production [42]. ROS induces signals for the opening of intercellular passages through which the leukocytes migrate. ROS and inflammatory mediators induce the expression of the vascular cell adhesion molecule 1 which functions as a scaffold for leukocyte migration and as an effective ligand for the integrin α_9_β_1_, which is highly and specifically expressed on neutrophils [43].

The interaction between neutrophils and endothelial cells further stimulates the release of granulocyte-macrophage colony-stimulating factor (GM-CSF) to recruit new neutrophils and increases the neutrophil lifespan by an auto-endocrine loop [44].

The tumor and mesenchymal cells in the TME express multiple chemokines and cytokines (e.g., CXCL1, CXCL2, CXCL5, CXCL6, IL1, IL1β, IL6, IL8, IL17, TNFα, and G-CSF), which are regulated by KRAS, SNAIL, and NOTCH signaling [45–47].

These ligands, involved in the mobilization and recruitment of neutrophils, are implicated in neutrophilia which is frequently observed in patients with cancer.

The constant draw on the bone marrow by tumor-secreted growth factors and TME-derived inflammatory mediators alters hematopoiesis and triggers the production of PMN-MDSC with aberrant effector functions and resistance to apoptosis (reviewed in [48]). PMN-MDSC adheres to and cross the endothelial barrier and follows the chemokine gradient to the target area [48–50]. The increasing PMN-MDSC and cytokine levels often also lead to splenic infiltration and splenic production of PMN-MDSC, which may eventually become the major source of these cells [51].

Upon downregulation of the main chemokine sensors CXCR1 and CXCR2 and upregulation of CXCR4, the majority of aged or senescent neutrophils exit target tissues, re-enter circulation, and return to the bone marrow, spleen, liver, and lungs where they undergo apoptosis and phagocytosis; this phenomenon is called reverse migration [52, 53]. Reverse migrating neutrophils are not able to migrate back to the target tissue; however, they do have a prolonged lifespan and increased capability of ROS production within the circulation. In murine models of infection, reverse migrating neutrophils were detected carrying live mycobacteria and viruses from the infected area to the draining lymph nodes [54, 55]. This could be a way to augment the adaptive immune response but may also represent a route for spreading the infection. Mechanistically, the reverse migration of neutrophils and migration of tumor cells are much alike [56] and there have been speculations if neutrophil reverse migration could support the dissemination of tumor cells to the sites of neutrophil clearance, which are indeed common sites of metastatic spread [6].

### Neutrophil activation and killing mechanisms

Neutrophils possess diverse weaponry to eradicate invading microorganisms and cells displaying non-self proteins. Upon adhesion to neighboring cells or ECM components, neutrophils are able to respond to a vast variety of environmental stimuli and express extreme functional plasticity. Maturation stage, age, cytokine- and PRR status, migratory capabilities, and NET formation dictate their functionality;[57] however, a comprehensive understanding of neutrophil heterogeneity in cancer is lacking [58].

Essential to tissue homeostasis, the phagocytic receptors on neutrophils directly sense distinct molecular patterns that provoke them to extend their membrane over the target followed by internalization, fusion with lysosomes, and protein degradation. Transformed host cells or invading pathogens display DAMP and PAMP that are intercepted by PRR on neutrophils, e.g., toll-like receptors and nucleotide-binding oligomerization domain (NOD)-like receptors that trigger the production of multiple cytokines and chemokines such as TNF, interferon-gamma (IFNγ), IL1, IL3, IL8, CXCL1, CXCL2, CXCL4, and CXCL5 as well as G-CSF, GM-CSF and leukotriene B_4_. Together, these signaling molecules cause a massive swarming of neutrophils to the target areas [59].

In proximity to T cells and following the exposure to specific cytokines like GM-CSF, IFNγ, and IL3, neutrophils are able to express the major histocompatibility complex II and acquire antigen-presenting phenotypes. They may even express co-stimulatory T cell receptor molecules and assist in the activation of T cells [60].

Like other innate immune cells, neutrophils kill their target by detecting activating ligands on target cell membranes and release of cytotoxic contents (proteases and ROS) from their granules [61].

Further, neutrophils are capable of mediating antibody-dependent cellular cytotoxicity by recognition of antigenic epitopes by immunoglobulin Fc receptors [62]. The ligation of neutrophil FcγRIIA or FcγRIIIB immunoglobulin-family receptors triggers the production and release of ROS such as hydrogen peroxide, hypochlorous acid, and various enzymes such as myeloperoxidase, elastase, and matrix metalloproteinases (MMP) that damage the target cell membranes, nucleic acids, and proteins. Meanwhile, the neutrophils themselves are endogenously protected from the oxidative stress they cause. In cancer cells, the lethal effects depend on the expression of the transient receptor potential channel subfamily M member 2, an H_2_O_2_-dependent Ca^2+^ channel, whose activation leads to a lethal influx of calcium ions [63]. Indeed, in a mouse model of breast cancer, H_2_O_2_ production by neutrophils inhibited metastatic seeding in the lungs [64]. Furthermore, ROS stimulates the production of proinflammatory cytokines (TNFα and CXCL2) and activates the release of proteases and the formation of NET [65].

As ROS are unstable oxygen-containing molecules with extreme reactivity due to an unpaired electron, excessive or long-term ROS production can cause significant tissue damage and may contribute to carcinogenesis due to oxidative DNA damage and genetic instability. Further, ROS inhibit T-cell differentiation and activation and increase T-cell death influencing the expression of *BCL2* and *FASLG* that are involved in apoptosis [66]. In addition, the neutrophil killing mechanisms involve trogocytosis, which is a direct mechanical disruption of the target cell plasma membrane and transferal of membrane fragments by endocytosis into the neutrophil. This mechanism is induced by target cell antibody coverage and directly augments neutrophils’ killing capacity [67] and may influence the immunotherapeutic efficacy.

### NET formation (NETosis)

**Fig. 2:**
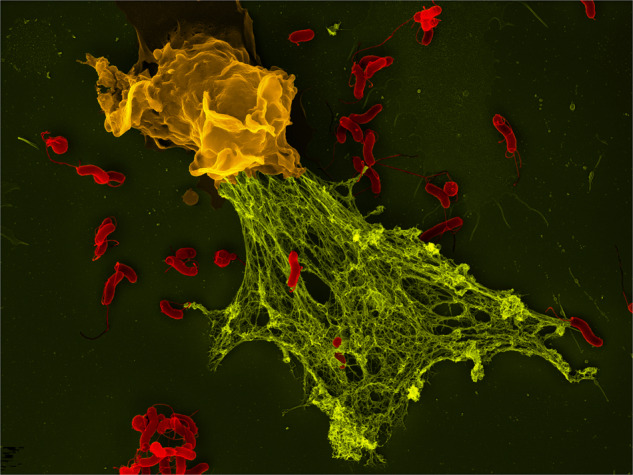
The neutrophil extracellular trap. A collapsing neutrophil has expelled its web to trap bacteria (Photo and permission by Volker Brinkmann, Max Planck Institute for Infection Biology, Berlin, Germany).

Neutrophils can expel web-like structures of protein-covered nucleic acids called as the NET, which are known to immobilize and neutralize pathogens (Fig. 2). Neutrophils are able to sense the size of a target and selectively release the NET in response to abnormal cells and large pathogens, but not in response to smaller targets such as single bacteria [68].

However, in cancer, NET may also trap CTC, promote extravasation, awaken dormant cancer cell, and prevent the physical interaction between cytotoxic immune cells and tumor cells [69].

Mechanistically, antigens triggering PRR and ligating Fc receptors cause an active fusion of the nuclear and cytoplasmic membranes of the neutrophils. This fusion results in the extracellular release of DNA filaments on which vast amounts of neutrophil-derived enzymes such as neutrophil elastase, myeloperoxidase, and cathepsin G get bound. The expulsion of NET results in immediate neutrophil cell death due to plasma membrane rupture; however, neutrophils may also release NET along with exocytosis of granules without dying and without the DNA loss having any impact on their lifespan and ability to phagocytose pathogens [70].

A systematic review analyzing the role of NETosis in disease progression in patients with colorectal cancer (CRC) revealed that the level of NET in the peripheral blood was associated with reduced recurrence-free survival [71]. Furthermore, NET formation due to surgical inflammation and complications facilitated tumor growth and outgrowth of tumor cells in pre-metastatic niches [71, 72].

Notably, in neutrophils derived from patients with hepatocellular carcinoma (HCC), NET trapped HCC cells, induced resistance to apoptosis, and enhanced invasiveness by internalization of the genetic material from NET into the trapped HCC cells [73].

The uncontrolled NET formation has deleterious consequences in liver, lung, and kidney damage and in cancer-associated vascular thrombosis which is observed in 20% of the patients with cancer [74].

Citrullinated histone H3 is a specific biomarker for NET formation. An elevated level of citrullinated histone H3, a key step in NET formation, is associated with increased short-term mortality in patients with cancer [75]. The H3 citrullination is a post-translational epigenetic conversion of histone arginine to citrulline, that loosens the tightly wound chromatin structure and promotes gene transcription.

Peptidyl-arginine-deiminase 4 (*PAD4), the* enzyme responsible for the conversion of arginine to citrulline and the citrullination of histones, is required for *NET* formation. In murine cancer models, PAD4 inhibitors inhibited NET, reverted the CD8^+^ T-cell exclusion, and improved the efficacy of immune checkpoint inhibitors (ICI) [76]. Although this enzyme inhibition offers a novel mode for targeted treatment, no PAD4 inhibitor has yet reached clinical trials.

Additionally, arginine deaminase and histone deacetylase inhibitors that block citrulline production and transcriptional repression are under evaluation as new targets in clinical trials [77, 78]. The novel drugs under investigation target important pathways involved in cell death, angiogenesis, and DNA repair.

## Prognostic value of neutrophil-to-lymphocyte ratio and circulating PMN-MDSC

Considered a surrogate marker of inflammation status and adaptive immune surveillance, neutrophil-to-lymphocyte ratio (NLR) gauges the balance between the two. Even though a direct association between NLR and prognosis cannot be made for all types of cancers, a pre-treatment NLR within the top 20^th^ percentile of any type of cancer is significantly associated with poor overall survival (OS) and progression-free survival (PFS) [79].

Further, elevated NLR is associated with low rates of response to ICI across multiple solid cancers including non-small cell lung cancer (NSCLC) [80], breast cancer [81], metastatic melanoma [82], prostate cancer [83], CRC [84], PDAC [85], renal cell carcinoma (RCC) [86], HCC [87], cholangiocarcinoma [88], ovarian cancer [89], and sarcoma [90]. Neutrophilia with an NLR ≥4 correlates with inferior disease-free survival (DFS) and OS in patients with mesothelioma, PDAC, RCC, and CRC as well as cancer-associated thrombosis [91–93].

An elevated NLR might be associated with a higher frequency of tumor-infiltrating neutrophils as demonstrated in patients with pancreatic cancer [94]. however, a general association between the NLR and the extent of neutrophil infiltration cannot currently be made or extrapolated to all cancer types.

In lung cancer, although the surgical resection was followed by a reduction in peripheral neutrophil counts, preoperative levels of circulating neutrophils and TAN were positively correlated with tumor burden and independently associated with a worse OS (*p* = 0.002) [95]. Likewise, a high neutrophil-to-CD8^+^ T cell ratio correlated to poor DFS and OS in patients with resectable esophageal squamous cell carcinoma [96].

In laryngeal cancer, there was a significant positive association between high NLR values, programmed cell death-ligand 1 (PD-L1) combined positive score <1 (indicating the number of *PD-L1* positive cells in relation to total tumor cells), T count rate <30%, and poor outcome highlighting the relationship between circulating immune cells and the TME characteristics [97].

In 2,280 patients with CRC undergoing primary tumor resection, both the NLR and the lymphocyte-monocyte ratio (LMR) were monitored preoperatively and postoperatively. Consistently low NLR and LMR were associated with vastly improved median OS compared to a high NLR and LMR at either interval [98]. Moreover, in a cohort of 1,744 patients undergoing curative CRC resection, the NLR was the only independent prognostic factor for poor OS and cancer-specific survival and superior to both the LMR and platelet-to-lymphocyte ratio [99].

## PMN-MDSC^TME^

In concert with tumor-derived chemokines (e.g., CXCL1, CCL2, and INFγ), CXC chemokines produced by tissue-resident immune cells promote the accumulation of neutrophils within the TME. Here, G-CSF, GM-CSF, transforming growth factor β (TGFβ), TNFα, IFNγ, IFNβ, and IL17 overexpressed by tumor cells, cancer-associated fibroblasts, and TAM drive the phenotypic switch that promote the reprogramming of neutrophils [6] and upregulate the immunosuppressive capacity of PMN-MDSC arriving from the circulation [18]. It has been demonstrated in murine models that PMN-MDSC^TME^ share similar surface markers with circulating PMN-MDSC including CD11b^high^, CD15^high^, CD16^high^, CD66b^high^, HLA-DR^−^, Arg1^high^, and LOX1^high^ [20, 100, 101]. Nevertheless, it is important to note that there is remarkable plasticity and phenotypic range between neutrophils and PMN-MDSC^TME^.

Single-cell RNA sequencing has confirmed the co-existence of classical PMN and PMN-MDSC in tumors in which PMN-MDSC appeared very early in tumor development and remained the most abundant PMN population [18].

The PMN-MDSC^TME^ overexpresses a variety of pro-tumorigenic cytokines, chemokines, and adhesion molecules and apart from ROS, the cytoplasmic granules contain a variety of enzymes (myeloperoxidase, elastase, and MMP such as collagenase [MMP8] and gelatinase B [MMP9]). MMP remodels the ECM to promote tumor cell migration (by degradation and realignment of collagen fibrils) and accelerate angiogenesis, thus counteracting the effects of anti-angiogenic molecules (reviewed in [6]).

**Fig. 3:**
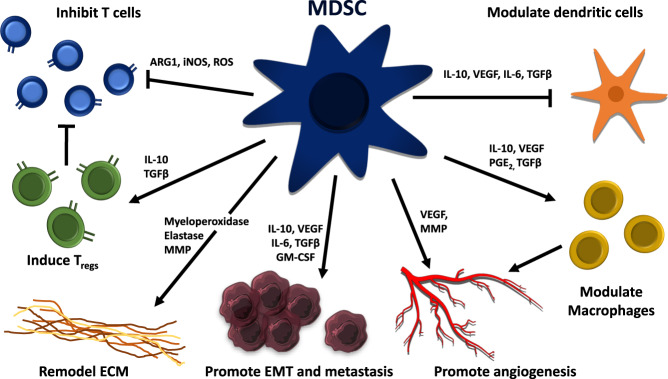
An overview of malignancy-associated mechanisms promoted by myeloid-derived suppressor cells. MDSCs inhibit CD4 and CD8 T cells while inducing regulatory T cells (Tregs). Through matrix metalloproteinases, MDSC promotes angiogenesis and enhances metastasis through anti-inflammatory mediators such as TGF-β and IL-10. Through IL-10, MDSCs also modulate dendritic cells and macrophages. ARG1 Arginase-1, ECM extracellular matrix, EMT epithelial–mesenchymal transition, INOS inducible nitric oxide synthetase, MMP metalloproteinase, GM-CSF granulocyte/macrophage colony-stimulating factor, PG prostaglandin E, VEGF vascular endothelial growth factor.

Proteases, elastase, and MMP derived from PMN-MDSC NET induce cell motility and proliferation of dormant tumor cells by the activation of integrin α3β1 signaling pathways [35, 102]. Along with tissue-resident stromal cells, PMN-MDSC also supports the formation of pre-metastatic niches as well as the colonization and outgrowth of disseminated tumor cells [103, 104] (Fig. 3).

PMN-MDSC are able to metabolize cysteine, L-arginine, and L-tryptophan which are essential for T cell functions. The lack of these compounds drives the differentiation of CD4^+^ T cells into regulatory T cells and inhibits T cell proliferation and T cell receptor assembly and thus rendering T cells anergic and unresponsive to antigen-specific stimulation. Further, nitration of CCL2 by MDSC-derived nitric oxide, prevents the infiltration of T cells into the central tumor confining them to the peripheral stroma [105].

In a mouse lung cancer model and in a *KRAS*-mutant human lung adenocarcinoma cell line (A549), elastase-induced degradation of insulin receptor substrate 1 leads to increased proliferation of tumor cells in both settings [106]. Furthermore, *CCL17*—a downstream mediator of GM-CSF and recruiter of immunosuppressive regulatory T cells—was found to be one of the most strongly upregulated genes among PMN-MDSC^TME^ that correlated with disease progression and poor prognosis [107].

Several studies (that use the term TAN without distinguishing between classically activated neutrophils and pathologically activated PMN-MDSC^TME^) have shown a correlation between TAN levels and poor prognosis in PDAC, HCC, melanoma, gastric cancer, RCC, and gliomas [93]. Variations in prognostic significance of TAN may be explained by variations in the geographical distribution of TAN within the TME, with intra-tumoral TAN having a stronger association with metastasis and poor outcome than peritumoral localization. This was most dramatically demonstrated in RCC, head, and neck squamous cell carcinoma, and lymphoma with hazard ratios for OS >2 [28, 93]. In CRC, TAN may predominantly be found at the invasive front and in the presence of tumor budding and marked TGFβ expression, both predicting a poor prognosis [108]. Notably, a meta-analysis of 18,000 tumor tissue samples revealed intra-tumoral PMN-MDSC^TME^ gene expression signatures to be the strongest predictor of poor survival across all cancer types [17]. Overall, the identification of PMN-MDSC^TME^ offers a wide scope in furthering our understanding of their functions and improving diagnostic and treatment trajectories.

### Neutrophils and metastases

Precisely how reverse migrating neutrophils eventually could support tumor cell migration is currently unknown. However, a pre-clinical study demonstrated that lipid-laden PMN-MDSC served as an energy source that fueled CTC during migration [19].

In a murine lung cancer model, cyclooxygenase (COX) and PGE2 pathways promoted mesenchymal cells in the TME to increase the expression of genes (*Hilpda* and *G0s2)* associated with reprogrammed lipid metabolism in PMN-MDSC to increase the uptake and storage of lipids. The lipid-laden neutrophils served as an energy source to fuel metastatic tumor cells, augmenting their proliferative and metastatic capacity [109].

In another murine study, PMN-MDSC selectively and significantly upregulated the fatty acid transporter protein 2 (FATP2) and the uptake of free fatty acids [105]. Through STAT5, GM-CSF controlled the expression of the FATP2-encoding gene *SLC27A2*, and an increased turnover of PMN-MDSC even reinforced lipid transfer. Interestingly, inhibition of FATP2 by lipofermata showed a reduction in tumor size in various murine models [21].

**Fig. 4:**
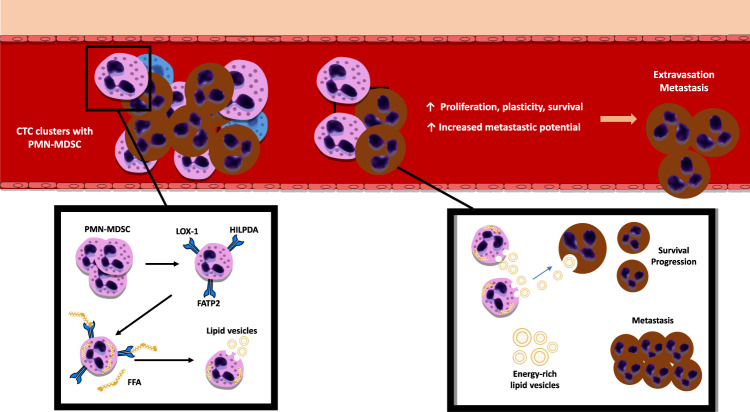
PMN-MDSC travel in clusters with CTC and stromal cells in the bloodstream. PMN-MDSC fuel the proliferation and survival of CTC by providing them with energy-rich lipid vesicles. The proximity of PMN-MDSC to CTC promotes the plasticity of CTC and increases their metastatic potential. In the TME, PMN-MDSCs accelerate their own lipid transfer, uptake, and storage of lipids through increased expression of FATP2, HILDPA, and LOX1 proteins. *CTC* circulating tumor cell, FATP2 fatty acid transporter protein 2, FFA free fatty acids, HILDPA hypoxia-inducible lipid droplet-associated protein, LOX1 lectin-like oxidized low-density lipoprotein receptor.

Traveling in clusters with CTC, PMN-MDSC supports cell cycle progression and survival of CTC by delivering energy-rich lipid vesicles [19, 109] (Fig. 4). Confirmed by high levels of Ki67 in CTC from neutrophil clusters, it was demonstrated that the proximity to neutrophils promoted CTC proliferation and plasticity within the circulation, and conferred CTC from these clusters with an increased metastatic potential [19]. In invasive breast cancer, circulating clusters of tumor cells and leukocytes are more prone to resulting in metastases than solitary CTC and thus lead to a significantly worse PFS [110].

## Targeting PMN-MDSC

It is now clear that PMN-MDSC induce immunosuppression and escape of tumor cells from host immunity. As the impact of neutrophils on anticancer therapy is unraveled, the immune profiles of the peripheral blood and TME are receiving ever-increasing interest from researchers. The role of PMN-MDSC in directly facilitating cancer cell migration and metastasis is also evident but the knowledge of the molecular mechanisms involved is still limited. At the same time, it is also not apparent in what phase of the patient trajectory the interventions targeting PMN-MDSC would have the greatest impact on curative treatment strategies in relation to surgery (perioperative strategies) or in stage IV cancer either as monotherapy or as adjuncts to other local or systemic treatments.

Circulating PMN-MDSC, as well as PMN-MDSC^TME^, can induce resistance to DNA-alkylating agents (gemcitabine and 5-fluorouracil) through exosomal delivery of miRNA to tumor cells to augment the repair of double-stranded breaks in the DNA [111, 112]. In addition, they confer resistance to ICI by increasing the expression of immune checkpoint proteins and tyrosine kinase inhibitors (VEGF- and mTOR inhibitors) through VEGF receptor, IL17, and PI3K/platelet-derived growth factor receptor (PDGFR) signaling [113].

Depletion of neutrophils is currently only achievable by extracorporeal granulocytapheresis, which has been used in selected cases of non-malignant disease [114]. However, the interruption of a number of signaling pathways that may represent treatment targets has been identified and pursued in drug development [115]. In the following sections, we will present the major therapeutic strategies areas involving targeting PMN-MDSC.

### Immunotherapy

Immunotherapy includes ICI that revitalizes dysfunctional T cells, adoptive cell transfer using CD8^+^ T cells equipped with synthetic chimeric antigen receptors (CAR-T) that enhance T cell functionality, and antibody-based therapies targeting cancer-specific antigens.

Despite showing positive responses initially, immunotherapies may lack a long-term effect and cause side effects that may lead to their discontinuation; this emphasizes the need for the development of long-lasting and safer therapeutic approaches.

A major challenge to the success of ICI therapy is the low levels of infiltrating lymphocytes in solid cancers (e.g., PDAC, triple-negative breast cancer, and CRC) [116]. TAN may directly inhibit effector T cell functions, contribute to immune exclusion, and recruit regulatory T cells (reviewed in [117]).

Targeting the tumor-promoting or tumor-protecting functions of PMN-MDSC may increase the efficacy of ICI and even expand ICI indications. Especially drugs that may influence PMN-MDSC-mediated immune exclusion, NET-mediated ICI resistance, NET-related thrombosis, and NET-mediated side effects to ICI therapy have been attractive prospects [117].

In HCC, circulating PMN-MDSC had a substantial PD-L1 expression, and the expression was even higher in the PMN-MDSC^TME^ [118] suggesting that PMN-MDSC are powerful players in T-cell exhaustion and important targets for anti-programmed cell death 1 (PD1) and/or anti-PD-L1 antibodies.

In laryngeal cancer, a PD-L1 CPS ratio ≥1 and a TIL count ≥30% within the TME were positively associated with higher DFS and reduced recurrence [97].

Anti-PD1 antibody therapy has become the first-line therapy for most patients with NSCLC either as monotherapy for PD-L1-high tumors or in combination with conventional chemotherapy for PD-L1-low tumors. Tissue analyses of patients with NSCLC demonstrated a subgroup of patients in which the presence of high numbers of PMN-MDSC was most likely responsible for preventing CD4^+^ and CD8^+^ T cell infiltration. These patients were resistant to ICI therapy and the CD8^+^ T cell/neutrophil ratio within the tumor stroma could effectively distinguish between responders and non-responders [119].

Preclinical studies point towards synergistic effects of the epigenetic modifiers entinostat (histone deacetylase inhibitor) and 5-azacytidine (DNA methylation blocker) in combination with ICI [120].

Currently, massive clinical efforts on immune profiling have been undertaken, but very few targeted MDSC therapeutics in combination with ICI have reached the clinic.

**Fig. 5:**
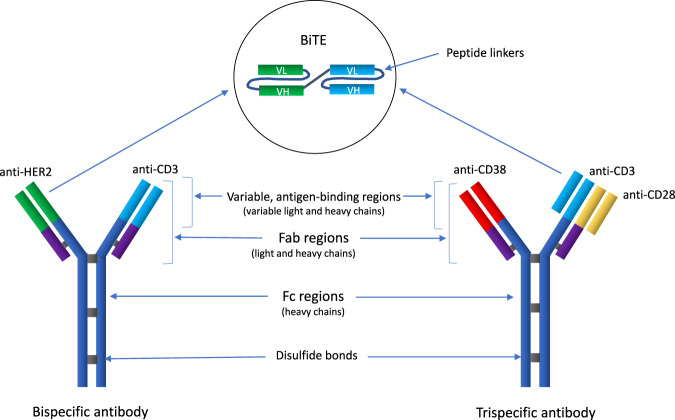
Schematic examples of bi- and trispecific antibodies and cell engager molecules. Bi- and tri-specific antibodies are engineered molecules designed to simultaneously bind two or three different targets, respectively. They are recombinant proteins with variable Fab regions or fragments of light chains. Novel antibodies under current clinical investigation target either one or two T-cell receptors (e.g., CD3 and CD28) and a tumor-specific (and stably expressed) antigen (e.g., CD19, CD38, HER2) with limited distribution in normal tissue to reduce off-target effects. CD3 binding induces T-cell stimulation and drives the T cell to the tumor cell. CD28 binding mediates co-stimulatory signals to fully activate the T cell receptor, reduce the release of non-specific cytokines, and increase the expression of Bcl-xL that blocks T-cell apoptosis. When the antibody anchors the T cell to the tumor cell epitope, it forms a synapse that leads to cytotoxic cytokine release, target cell killing, and T-cell proliferation. Through serial lysis, individual T cells are able to induce multiple tumor cell killings. Bispecific engager molecules: The variable antigen-binding regions of the heavy and light chains can be fused together to form a single-chain variable fragment (scFv), which is only half the size of the Fab fragment but still retains the specificity of the parent antibody. These molecules are recombinant, scFv fusion protein constructs comprised of the antigen-binding regions (variable heavy and light chains) of two antibodies bound together by linker peptides (usually Ser-Gly peptide linkers). An engager molecule binds with e.g., CD3 on T cells (bispecific T cell engager—a BiTE) or CD16 on NK cells (bispecific killer-cell engager—a BiKE). The other arm binds to a tumor-associated antigen (e.g., HER2). Tri- and tetra-specific killer-cell engagers (TriKEs and TetraKEs) have been designed and are currently under clinical evaluation. Fab region the fragment antigen-binding region that binds to antigens is composed of one constant and one variable domain of each of the heavy and the light chain. FC *region* the fragment crystallizable region is the tail region of the antibody that interacts with cell surface Fc receptors. VL variable light chain, VH variable heavy chain.

Developers of antibody therapies search to expand the possibilities of bi- and tri-specific monoclonal antibodies and cell engager constructs (Fig. 5) that bind different targets simultaneously, such as securing the immunological synapse between effector cells and tumor cells whilst blocking the disease-related pathways. So far, only one such antibody (blinatumomab) has been approved by FDA for the treatment of lymphoblastic leukemia. As with CAR-T therapy, the progress is slow for antibodies targeting solid tumors due to the lack of unique tumor antigens. Moreover, several trials in the pipeline have been halted because of the futility or toxicity of the products. The safety issues are currently being addressed by T cell engager technologies, where specific tumor proteases unmask and activate the antibody pro-drugs [121].

## Chemokine inhibitors

PMN-MDSC actively communicate with cancer cells through inflammatory mediators and growth factors such as IL8 [122], IL17a [123], CCL2 [124], TNFα [125], BV8, VEGFα [126], and TGFβ plays important roles in various aspects of tumor cell dissemination, invasion, and angiogenesis. High IL8 expression has been shown to correlate negatively with T-cell markers and IFNγ-dependent gene signatures in the TME leading to the conclusion that mechanisms of T-cell exclusion and immunosuppression involve PMN-MDSC [19, 127]. Anti-IL8 antibodies are currently being evaluated as monotherapy or in combination with ICI in several clinical trials (Table 1).

**Table 1:** Ongoing and completed clinical trials evaluating the interventions involving chemokine and cytokine targetting to affect PMN-MDSC functionality.

Trial number and status	Cancer type	Investigational drug	Target	Mechanism	Preliminary results and adverse events
NCT02536469/completed	Locally advanced solid tumors	BMS-986253	IL8	Inhibition of IL8 using a monoclonal antibody	No ORR. 11 patients (73%) had SD. Grade 1 treatment-related AE occurred in five patients (33%). Two patients receiving 32 mg/kg dose had grade 2 fatigue, hypophosphatemia, and hypersomnia.
NCT03400332/ongoing	Advanced cancers	BMS-986253 + nivolumab/+ Ipilimumab			NA
NCT04123379/ongoing	NSCLC, HCC	Nivolumab + BMS-986253 + BMS-813160	IL8 + CCR2/5		NA
NCT03473925/completed	Advanced/metastatic solid tumors	Navarixin (MK-7123) + Pembrolizumab	CXCR1/2	Allosteric inhibition of CXCR activation by its ligands IL8 and CXCL8, causes cancer stem cell apoptosis and may inhibit tumor cell progression.	NA
NCT04477343/ongoing	Pancreatic ductal Adenocarcinoma	SX-682 + nivolumab			NA
NCT03161431/ongoing	Metastatic melanoma	Arm A: SX-682 alone; Arm B: SX-682 + pembrolizumab			NA
NCT01861054/terminated as enrollment target was not reached	Early-stage breast cancer	Reparixin			NA
NCT02001974/completed	Metastatic breast cancer	Reparixin + paclitaxel			No grade 4-5 AEs or treatment-related serious AEs observed. No interactions between reparixin and paclitaxel. A 30% response rate was observed, with durable responses over 12 months in 2 patients.
NCT02370238/completed	Metastatic triple-negative breast cancer	Reparixin + paclitaxel			PFS similar across control and experimental arms. Serious AE occurred in 21.3% and 20% subjects in both groups.
NCT03177187/ongoing	Metastatic castration-resistant prostate cancer	AZD5069 + enzalutamide	CXCR2	Selective small-molecule inhibitor that has 100 times higher specificity for CXCR2	NA
NCT02472977/terminated due to lack of short-term efficacy	Pancreatic and small cell lung cancer	Ulocuplumab + nivolumab	CXCR4	Binds to CXCR4 and inhibits the binding of CXCL12 to CXCR4 and its subsequent activation, which may inhibit the tumor cell proliferation and migration.	NA
NCT01359657/completed	Multiple myeloma	Ulocuplumab + dexamethasone + lenalidomide/bortezomib			ORR, 50% (22/44) with 1 CR, 15 PR, 8 SD (mean 159 days). Grade 3 AE included thrombocytopenia, anemia, respiratory infection, lymphopenia, and neutropenia among others.
NCT01120457/completed	Acute myelogenous leukemia Diffuse large B-cell leukemia Chronic lymphocytic leukemia Follicular lymphoma	Ulocuplumab			NA
NCT03111992/completed	Multiple myeloma	Arm A: CJM112; Arm B: CJM112 + PDR001; Arm C: PDR001 + LCL161	IL17A	Prevents binding of IL17A to the IL-17 receptor and inhibits IL17A/IL-17R-mediated signaling and inflammation.	NA
NCT03293784/ongoing	Advanced melanoma	Cohort 1: certolizumab + ipilimumab + nivolumab; cohort 2: infliximab + ipilimumab + nivolumab	TNFα	Certolizumab (Fab fragment of monoclonal antibody) and Infliximab (IgG1κ monoclonal antibody) bind to soluble and membrane-bound TNFα and block its proinflammatory functions.	NA
NCT02906397/completed	HCC	Galunisertib (LY2157299) + stereotactic body radiotherapy	TGFβ	Oral small-molecule inhibitor of the TGFβ receptor I, kinase that downregulates the phosphorylation of SMAD2, blocks activation of the canonical pathway	NA
NCT03206177/ongoing	Ovarian/uterine carcinosarcoma	Galunisertib + paclitaxel/carboplatin			NA
NCT02734160/completed	Metastatic pancreatic cancer	Galunisertib + durvalumab			Limited clinical activity. 1 PR; 7 SD; DCR: 25%; OS: 5.72 months; PFS: 1.87 months. No dose-limiting toxicities observed.
NCT02423343/completed	Advanced refractory solid tumor recurrent NSCLC and HCC	Galunisertib + nivolumab			NA
NCT02452008/ongoing	Castration-resistant prostate cancer	Galunisertib + enzalutamide			NA
NCT02672475/ongoing	Metastatic AR^−^ triple-negative breast cancer	Galunisertib + paclitaxel			NA
NCT02688712/ongoing	Locally advanced rectal adenocarcinoma	Galunisertib + capecitabine + 5-fluorouracil + total specific mesorectal excision			NA

*AE* adverse events, *AR* androgen receptor, *CR* complete response, *CCR2/5* C-C chemokine receptor type 2/5, *CXCR1/2/4* C-X-C chemokine receptor 1/2/4, *CXCL12* C-X-C chemokine ligand 12, *DCR* disease control rate, *HCC* hepatocellular carcinoma, *IL* interleukin, NSCLC non-small cell lung cancer, *ORR* objective response rate, *OS* overall survival, *PFS* progression-free survival, *PR* partial response, SD stable disease, *SMAD2* Mothers against decapentaplegic homolog 2 protein, *TNFα* tumor necrosis factor α, *TGFβ* transforming growth factor β.

Chemokines receptors and their ligands play prominent roles in neutrophil trafficking. CXCR1/2 are highly expressed by neutrophils, and guide them to their dominant ligand IL8 and alternate ligands (CXCL1, CXCL2, CXCL5, and CXCL6) that are elevated in many cancers and regulated by various factors in the TME (as reviewed in [128]). Aiming to block tumor cell migration, the CXCR1/2 antagonists have begun to reach the clinic (Table 1). The CXCR1/2 inhibitors (reparixin, SX-682) have been in clinical trials in combination with paclitaxel for breast cancer (NCT01861054, NCT02370238, and NCT02001974); however, results have not been published yet. Similarly, an ongoing stage 2 clinical trial (NCT03473925) on advanced microsatellite-stable CRC, castration-resistant prostate cancer, and NSCLC is investigating NET formation during combination therapy with a CXCR1/2 antagonist (navarixin) and PD1 inhibitor (pembrolizumab).

The binding of CXCL12 (stromal cell-derived factor 1) to CXCR4 on tumor cells enhances proliferation, either via MAPK or PI3K/Akt pathways, and CXCL12 also recruits immunosuppressive cells as Tregs and MDSC that contribute to immune evasion [129]. The CXCR4 inhibitor ulocuplumab has been investigated in hematological malignancies (NCT01120457, NCT01359657). Notably, one study on ulocuplumab in PDAC and small cell lung cancer was terminated due to futility (NCT02472977).

To decrease or inhibit neutrophil recruitment to tumors, the CXCR1/2 antagonist reparixin is currently being tested in combination with paclitaxel as first-line therapy in patients with metastatic triple-negative breast cancer (NCT02370238). SX-682, another CXCR1/2 inhibitor is being investigated in pancreatic ductal adenocarcinoma (PDAC) and metastatic melanoma (NCT04477343 and NCT03161431), whereas the selective CXCR2 inhibitor AZD5069 is being evaluated in metastatic castration-resistant prostate cancer (NCT03177187).

IL17a is an immune and inflammatory mediator with multiple biological activities. IL17a is widely found in the inflammatory microenvironment of various tumors and is involved in tumor cell dissemination, chemotherapy resistance, and immunosuppression. In a study, where TAN was isolated from patients with gastric cancer, it was demonstrated that IL17a derived from these cells promoted the epithelial-mesenchymal transition through JAK2/STAT3 signaling. The use of a neutralizing antibody inhibited the TAN stimulated activities that also included enhanced migratory and invasive capabilities [130].

The effects of CJM112 (anti-IL17A and anti-IL17F) therapy on the PMN-MDSC compartment is currently being evaluated in multiple myeloma (NCT03111992; Table 1).

The role of anti-TNFα monoclonal antibodies such as infliximab in cancer is still debated. Anti-TNF therapy is currently used to treat ICI complications like colitis. In some cases, anti-TNF treatment seems to boost the ICI response without increased risk of later recurrence [131]. The efficacy of anti-TNF therapy in combination with ICI in advanced melanoma is being evaluated in an ongoing clinical trial (NCT03293784).

### TGF-β inhibitors

Overexpression of TGFβ by tumor cells is a major suppressor of both adaptive and innate immune responses, promoting all stages of tumor development in several cancer types [132]. Besides polarizing neutrophils into PMN-MDSC, TGFβ is also a strong neutrophil chemoattractant and its inhibition could potentially result in impaired neutrophil recruitment [133, 134]

Galunisertib is a novel TGFβ receptor 1 kinase inhibitor that was recently investigated in a phase I trial in combination with anti-PD-L1 in patients with recurrent and/or refractory metastatic pancreatic cancer; however, the clinical activity was limited. The investigators suggested that the combination could be more effective in an earlier line of treatment [135].

Multiple trials on TGFβ receptor inhibitors often in combination with ICI or standard therapies are ongoing (Table 1; ClinicalTrials.gov).

### Tyrosine kinase inhibitors (TKI)

Translocations, amplifications, and mutations activate many tyrosine kinases (TK) in cancer. TK is directly implicated in all disease stages and are major targets for drug discovery. Already, multiple TKI targeting EGFR, ALK, ROS1, HER2, NTRK, VEGFR, RET, MET, MEK, FGFR, PDGFR, and KIT have been developed and more than 40 drugs have gained FDA approval in cancer therapy [136].

The spleen tyrosine kinase (SYK) is especially important for the neutrophil Fc receptor and β2 integrin signaling and thus selective SYK inhibitors have been developed and are under investigation for hematological malignancies (See trial details in Table 2).

**Table 2:** Ongoing and completed clinical trials evaluating the interventions involving tyrosine kinase targetting to affect PMN-MDSC functionality.

Trial number and status	Cancer type	Investigational drug	Target	Mechanism	Preliminary results and adverse events
NCT03742258/ongoing	Diffuse large B-cell lymphoma	TAK-659 + cyclophosphamide + doxorubicin + prednisone + rituximab + vincristine sulfate	SYK + FLT3	TAK-659 is a dual Syk and FLT3 inhibitor that blocks the cell survival, proliferation, chemoresistance, and effects promoted by the TME through β2 integrin signaling (ref: https://jhoonline.biomedcentral.com/articles/10.1186/s13045-017-0512-1)	NA
NCT05028751/ongoing	Acute myeloid leukemia Relapsed acute myeloid leukemia Refractory acute myeloid leukemia	Lanraplenib + gilteritinib	SYK + FLT3	Lanraplenib: Syk inhibitor; gilteritinib: FLT3 inhibitor	NA
NCT03010358/completed	Relapsed chronic lymphocytic leukemia, small lymphocytic lymphoma, or non-Hodgkin lymphoma	Entospletinib (GS-9973) + Obinutuzumab	SYK + CD20	Entospletinib: SYK inhibitor; obinutuzumab is a anti-CD20 monoclonal antibody which mediates B-cell lysis through cellular cytotoxicity and phagocytosis	Entospletinib led to downregulation of pSTAT3 and MCL1 in CLL cells. 6-month combintion therapy led to reduced IFN-γ secretion in CD4^+^ T cells
NCT01799889/discontinued	Relapsed or refractory hematologic malignancies	Entospletinib	SYK	Entospletinib: SYK inhibitor	PFS: 13.8 months (95% CI, 7.7 months to not reached); ORR: 61% (95% CI, 44.5%-75.8%). SAE in 54/186 (29.0%) including dyspnea, pneumonia, febrile neutropenia, dehydration, and pyrexia. Common grade 3/4 AE included neutropenia (14.5%) and reversible ALT/AST elevations (13.4%).
NCT01796470/terminated for safety reasons	Relapsed or refractory hematologic malignancies	Entospletinib + idelalisib	SYK	Entospletinib: SYK inhibitor	ORR: 60% (CLL) and 36% (FL). Pneumonitis in 18% patients (severe in 11/12 cases and 2 deaths due to treatment-emergent pneumonitis)
NCT00446095/completed	B-cell lymphomas	Fostamatinib	SYK	Selective abrogation of the BCR signaling pathway leading to strong anti-inflammatory effects	ORR: 22% (5 of 23) for DLBCL, 10% (2 of 21) for FL, and 55% (6 of 11) for SLL/CLL. PFS: 4.2 months. Common AE included diarrhea, fatigue, cytopenias, hypertension, and nausea.
NCT01499303/completed	Diffuse large B-cell lymphoma	Fostamatinib	SYK		ORR: 2/21. Common AE included diarrhea, vomiting, pyrexia, neutropenia among others
NCT03246074/ongoing	Ovarian cancer	Fostamatinib + paclitaxel	SYK		NA
NCT01994382/ongoing	Non-Hodgkin lymphoma, chronic lymphocytic leukemia, small lymphocytic leukemia	Arm A: cerdulatinib; Arm B: cerdulatinib + rituximab	SYK + JAK 1/3	Dual kinase blocker of Syk and JAK 1/3. inhibits BCR- and IL4-induced downstream signaling in CLL.	NA

*AE* adverse events, *ALT* alanine aminotransferase, *AST* aspartate aminotransferase, *CLL* Chronic Lymphocytic Leukemia, *DLBCL* diffuse large B-cell lymphoma, *FL* follicular lymphoma, *FLT3* fms-like tyrosine kinase 3, *IL* interleukin, *JAK* Janus kinase 1, *MCL1* myeloid leukemia cell differentiation protein 1, *ORR* objective response rate, *PFS* progression-free survival, *SLL* Small Lymphocytic Leukemia, *STAT3* Signal transducer and activator of transcription 3, *SYK* spleen tyrosine kinase, *TME* tumor microenvironment.

On 21 March 2021, FDA approved lorlatinib (Lorviqua), a 3^rd^ generation small-molecule TKI, for anaplastic lymphoma kinase-positive NSCLC. Preclinical trials on PDAC and CRC models were recently conducted to study the effect of this molecule in other solid cancers with heterogeneous TME [21]. In PDAC, lorlatinib modulated neutrophil development and recruitment from the bone marrow through abrogation of G-CSF, GM-CSF, and CXCL1 signaling that reduced the number of TAN. Further, the amount of ECM and the size of PDAC tumors and metastases were reduced. Through elevated levels of tumor-infiltrating CD8^+^ T cells, lorlatinib increased the therapeutic response to anti-PD-1 treatment and improved survival. In the CRC arm of the trial, hepatic metastases were initiated by intrasplenic injections of organoid-derived CRC cells. Lorlatinib reduced the growth of metastases and the number of neutrophils in the lesions; however, there was no significant change in the number of metastases in the study group when compared to that in the control group [137].

In general, TKI may block several (and irrelevant) kinases and it is therefore critical to develop more selective antibodies since multi-targeting TKI can cause unnecessary off-target toxicities. The next-generation kinase inhibitors will probably show an improved selectivity and an ability to combat resistance mechanisms and maybe play a key role in immune signaling to complement immuno-oncology approaches [138]. Furthermore, interventions with kinase inhibitors before the onset of more advanced metastatic disease could be a significant step towards improving the efficacy and overall survival efficacy of kinase inhibitors.

### Prostaglandin E2 and COX inhibition

The COX enzymes (mainly COX2) degrade cell membrane phospholipids and trigger the release of PGE2. Apart from increased mitogenic signaling, genomic instability, and suppression of apoptosis, PGE2 release by PMN-MDSC and PMN-MDSC^TME^ represents an important mechanism of T-cell suppression.

Multiple preclinical trials have demonstrated the potential for COX2 inhibition to increase the efficacy of immunotherapy and several clinical trials on COX2 inhibitor and ICI are ongoing. One important caveat is that COX2 inhibitors are linked to GI bleeding and cardiovascular events, including death, meaning that the safety profile of COX2 in combination with ICI will have to be firmly defined prior to broader clinical use. A specific PGE2 receptor inhibitor, grapiprant, is currently tested in combination with ICI for microsatellite-stable CRC and NSCLC (Table 3).

**Table 3:** Ongoing and completed clinical trials evaluating the interventions involving COX2 and PGE2 targetting to affect PMN-MDSC functionality.

Trial number and status	Cancer type	Investigational drug	Target	Mechanism	Preliminary results and adverse events
NCT00652340/completed	Non-small cell lung cancer	Apricoxib/placebo + erlotinib	COX2	Blocks COX2 activity and reduces PGE2 levels to reduce resistance to EGFR inhibitor	ORR: 12% in both groups. OS in subgroup with >65 years of age had higher TTP and OS (12 months vs 4.1 months). Common AE included rash, diarrhea, fatigue, and nausea.
NCT03026140/ongoing	Colon carcinoma	Nivolumab + ipilimumab ± celecoxib	PD-L1 + CTLA4 + COX2	Blocking COX2 activity inhibits the T-cell suppression and therefore activates immune system and helps improve response to ICI	NA
NCT03926338/ongoing	Colorectal cancer	Toripalimab ± celecoxib	PD1 + COX2		NA
NCT03728179/ongoing	Advanced TIL-negative solid tumors	Low dose irradiation + nivolumab + ipilimumab or cyclophosphamide + aspirin/celecoxib	COX2		NA
NCT04188119/ongoing	Triple-negative breast cancer	Avelumab + aspirin + lansoprazole	PD-L1 + COX2		NA
NCT04348747/ongoing	Metastatic triple-negative breast cancer	Celecoxib + pembrolizumab + anti-her2 vaccine + recombinant interferon α2b + rintatolimod	PD1 + COX2	Rintatolimod, interferon alpha-2b and celecoxib to direct the immune cells to the cancer and maximize efficacy of pembrolizumab	NA
NCT03245489/ongoing	Recurrent/metastatic squamous cell carcinoma of head and neck	Pembrolizumab + clopidogrel + acetylsalicylic acid	PD1 + antiplatelet activity + COX2	Blocking COX2 activity inhibits the T-cell suppression and therefore activates immune system and helps improve response to ICI.	NA
NCT05041101/ongoing	Metastatic inflammatory breast cancer	Grapiprant + eribulin	EP4 (PGE2 receptor) + microtubule inhibition	Selective antagonism of EP4 receptor, one of the 4 PGE2 receptor subtypes. EP4 receptor mediates PGE2-elicited nociception.	NA
NCT03696212/terminated	Non-small cell lung cancer	Grapiprant + pembrolizumab	EP4 + PD1	Selective antagonism of EP4 receptor, one of the 4 PGE2 receptor subtypes and PD1/PD-L1 blockade	NA
NCT03658772/ongoing	Advanced or progressive MSS colorectal cancer	Grapiprant + pembrolizumab			

*AE* adverse events, *COX2* cyclooxygenase 2, *CTLA4* cytotoxic T-lymphocyte-associated protein 4, *EGFR* endothelial growth factor receptor, *ICI* immune checkpoint inhibitors, *ORR* objective response rate, *OS* overall survival, *PGE2* prostaglandin E2, *PD1* programmed cell death 1, *PD-L1* programmed cell death-ligand 1, *TTP* time to progression.

Whereas NET traps CTC and fuels the metastatic process, anti-inflammatory drugs like acetylic salicylic acid, an unselective, irreversible COX inhibitor, proved effective in inhibiting HCC metastasis in combination with DNase 1 through multiple mechanisms among others by blocking and digesting NET and abrogating the metastatic potential of trapped HCC cells. The use of well-known anti-inflammatory drugs in combinatory regimens is likely to be developed.

Recently, it was reported that the expression of FATP2 accelerates the uptake of triglycerides by PMN-MDSC and that lipid-laden PMN-MDSC plays critical roles in PMN-MDSC mediated T-cell suppression and supported migrating tumor cells. In vitro, FATP2-deficient neutrophils show low ability to suppress T-cell proliferation, and lipofermata, a FATP2-inhibitor, reduced tumor growth and increases sensitivity to anti-CTLA-4 immunotherapy. Nonetheless, lipofermata has not yet reached the clinic [21].

## Discussion

Exhibiting marked heterogeneity and functional versatility, neutrophils are the protagonists during immune responses. Although they are crucial to our health, pathological activation and impaired resolution of neutrophils can cause chronic illness and cancerous tissue transformation. Malignancies are often associated with the expansion of the neutrophil compartment and cancer-mediated neutrophilia has been demonstrated in several cancers. Displaying immense plasticity and cross-talk with other cell types, escalating levels of PMN-MDSC tend to accumulate in the circulation and in the TME regardless of the tumor type or stage.

The increasing numbers of PMN-MDSC correlate with poor outcomes in several solid cancers such as RCC, melanoma, CRC, HCC, head and neck cancer, and NSCLC [17]. The data from preclinical tumor models underlines the significant therapeutic effects of eliminating PMN-MDSC, which make PMN-MDSC extremely relevant targets. Although their turnover and exceptional plasticity make PMN-MDSC formidable adversaries, several therapeutic strategies targeting PMN-MDSC functions are being investigated clinically (Tables 1–3).

PMN-MDSC use multiple mechanisms to suppress anticancer immunity and their depletion as a treatment modality seems like an appealing concept; however, depletion of PMN-MDSC is not feasible in humans currently. On the brighter side, a number of signaling pathways that may represent potential treatment targets have been identified. The identification of specific molecular markers of PMN-MDSC represents a significant advancement in the classification of these cells. Ongoing improvements in sequencing techniques and proteomics will further guide us in the quest for finding novel immunotherapeutic targets.

In addition to ICI revitalizing the tumor-infiltrating cytotoxic immune cells, engineered biologics such as adoptive immune-cell therapies, T-cell engagers, and multi-specific antibodies are likely to reach the clinic in the near future. To further improve outcomes, targeting PMN-MDSC may be key for next-generation immunotherapies.

To address the versatility of PMN-MDSC, the cross-talk with cell populations within the TME, and their potential reprogramming, future studies should investigate tissue-resident stromal and immune cells as well as the origin of PMN-MDSC outside of the tumor bed, i.e. the bone marrow and spleen.

When it comes to the treatment of solid cancers, surgical resection remains the therapeutic mainstay. However, a substantial number of patients experience disease recurrence after radical surgery. Paradoxically the surgical injury may enhance the dissemination of tumor cells and the growth of residual cancer tissue by suppression of innate and adaptive immunity as well as accelerating the expansion of MSDC. Preclinical and clinical studies indicate that the expansion and levels of MDSC correlate with the disease stage and the magnitude of the surgery-induced inflammatory response (surgical stress response). However, results are not univocal and conflicting evidence exists [139–141]

Apart from the suppression of anticancer immunity, MSDC supports the formation of pre-metastatic niches (fertilize the soil) and reinforces the survival, proliferation, and extravasation of CTC (sow the seeds). Evidence is scarce regarding the direct impact of surgery on MDSC, however, research points towards MDSC as the central players orchestrating the diverse and complicated machinery of postoperative metastasis formation [16]. The perioperative period should attract attention with the aim of targeting MDSC and identifying the optimal window of opportunity.

The shift in perspective in which we use translational research and focus on molecular alterations in cancer has opened the door for individually targeted therapies. Nonetheless, we need an integrated approach to look at the combined interactions between tumor cells and immune cells similar to that shown by Pelka et al. [142].

Subsequently, we must assimilate advanced datasets to evaluate the heterogeneity of immune responses and tumor profiles across patients and assess the impact on disease development in order to tailor optimal personalized therapies.

## Data Availability

All data presented in the current study are publicly available in the MEDLINE database in accordance with the reference list.
